# A Novel Homozygous T-Cell Immune Regulator 1 (TCIRG1) Stop-Gain Variant Causing Malignant Infantile Osteopetrosis

**DOI:** 10.7759/cureus.111709

**Published:** 2026-06-29

**Authors:** Asmae Baaziz, Asmaa Mdaghri Alaoui

**Affiliations:** 1 Dysmorphology Unit, Pediatrics P2 Department, Children's Hospital, Ibn Sina University, Rabat, MAR

**Keywords:** bicytopenia, cerebral atrophy, consanguinity, hematopoietic stem cell transplantation, malignant infantile osteopetrosis, novel variant, tcirg1, whole exome sequencing

## Abstract

Malignant infantile osteopetrosis (MIOP) is a rare life-threatening autosomal recessive disorder characterized by defective osteoclast-mediated bone resorption, most commonly caused by pathogenic variants in the TCIRG1 gene. Without hematopoietic stem cell transplantation (HSCT), the disease is associated with severe morbidity and early mortality. We report a 15-month-old Moroccan girl born to first-cousin consanguineous parents who initially presented during infancy with failure to thrive, persistent bicytopenia, developmental delay, axial hypotonia, divergent strabismus with nystagmus, and facial dysmorphism. Skeletal radiographs demonstrated diffuse osteosclerosis, obliteration of medullary cavities, and characteristic Erlenmeyer flask deformities, strongly suggestive of MIOP. Whole-exome sequencing identified a novel homozygous pathogenic variant in T-cell immune regulator 1 (TCIRG1) (NM_006019.4:c.1897C>T; p.Gln633Ter). The variant is absent from the gnomAD database and was classified as pathogenic according to the American College of Medical Genetics and Genomics/Association for Molecular Pathology (ACMG/AMP) criteria. To the best of our knowledge, this variant has not previously been reported in affected individuals. The patient is currently undergoing evaluation for HSCT. This case expands the mutational spectrum of TCIRG1-associated MIOP and highlights the importance of early genomic testing in infants from consanguineous families presenting with unexplained cytopenias, growth failure, and characteristic skeletal abnormalities.

## Introduction

Osteopetrosis ("marble bone disease") comprises a clinically and genetically heterogeneous group of rare inherited disorders characterized by impaired osteoclast-mediated bone resorption, resulting in increased bone density despite structurally fragile bone [1]. Malignant infantile osteopetrosis (MIOP; OMIM #259700) represents the most severe form of the disease and is inherited in an autosomal recessive manner, with an estimated incidence of approximately one in 250,000 live births [2]. Without curative treatment through hematopoietic stem cell transplantation (HSCT), MIOP is associated with high mortality in early childhood due to bone marrow failure, recurrent infections, and progressive neurological complications [3].

Biallelic pathogenic variants in T-cell immune regulator 1 (TCIRG1), encoding the a3 subunit of the vacuolar H+-ATPase (V-ATPase) proton pump, account for approximately 50-60% of MIOP cases [4]. Loss of TCIRG1 function disrupts acidification of the osteoclastic resorption lacuna, preventing dissolution of hydroxyapatite crystals and leading to defective bone remodeling. Progressive skeletal sclerosis results in obliteration of medullary cavities, impaired hematopoiesis, extramedullary hematopoiesis, hepatosplenomegaly, cranial nerve compression, and severe growth impairment [4,5].

Affected infants typically present within the first months of life with failure to thrive, cytopenias secondary to bone marrow compromise, hepatosplenomegaly, developmental delay, visual impairment due to optic nerve compression, and characteristic radiological findings of generalized osteosclerosis [6,7]. Neurological manifestations may develop as a consequence of progressive narrowing of cranial foramina and remain a major determinant of long-term morbidity.

Numerous pathogenic variants have been identified in TCIRG1, including missense, nonsense, splice-site, frameshift, and deletion variants. Most are predicted to result in loss of protein function and are associated with the severe infantile phenotype [1]. The marked allelic heterogeneity of TCIRG1, together with the predominance of private family-specific variants, highlights the importance of comprehensive molecular testing for definitive diagnosis and genetic counseling. Data from North African populations remain limited despite the relatively high prevalence of consanguineous marriages.

We report a Moroccan girl from a consanguineous family with MIOP caused by a novel homozygous TCIRG1 stop-gain variant, c.1897C>T (p.Gln633Ter). This variant has not previously been reported in affected individuals. This case expands the mutational spectrum of TCIRG1-associated osteopetrosis and further emphasizes the value of genomic testing in the diagnosis of rare skeletal disorders.

## Case presentation

Family history and background

The proband is a female infant born to a 28-year-old primigravida mother with no significant personal medical history. The parents are first-cousin consanguineous. The patient has a nine-year-old brother who underwent clinical examination and was found to be phenotypically unaffected, with no features suggestive of osteopetrosis or related skeletal abnormalities, and no similarly affected individuals have been identified in the extended family.

The pregnancy was uneventful and carried to term. Delivery was performed by elective cesarean section. Birth weight was 2,285 g, and an immediate neonatal cry was reported. The neonatal period was unremarkable.

The first clinical concerns arose at approximately three months of age, when recurrent postprandial vomiting and poor weight gain were noted. At 4.5 months of age, the infant was hospitalized for evaluation of bicytopenia. Laboratory investigations revealed normocytic normochromic anemia (hemoglobin: 7.7 g/dL; reference range: 11.5-16.5 g/dL), thrombocytopenia (45,000/mm³; reference range: 150,000-400,000/mm³), and leukocytosis (23,000/mm³; reference range: 4,500-13,000/mm³), with lymphocytic predominance.

Bone marrow aspiration demonstrated a hypercellular regenerative marrow with trilineage hematopoiesis, no dysplastic features, and no evidence of hematological malignancy or infiltrative process. These findings were consistent with myelophthisis secondary to osteopetrotic bone encroachment rather than a primary hematological disorder. The patient received platelet transfusion support. Empirical corticosteroid therapy (prednisolone 5 mg/day) was initiated for three months before the molecular diagnosis was established, based on an initial working diagnosis of immune-mediated thrombocytopenia, given the persistent bicytopenia and absence of a definitive etiology at that time. No sustained hematological improvement was observed, and corticosteroid therapy was subsequently discontinued following molecular confirmation of malignant infantile osteopetrosis.

Clinical examination

At the time of genetic evaluation (15 months of age), anthropometric measurements were as follows: weight 5.6 kg (<3rd percentile), length 61 cm (<3rd percentile), and head circumference 46 cm (>97th percentile), according to WHO Child Growth Standards. These findings confirmed severe failure to thrive associated with macrocephaly.

General examination revealed an alert but hypotonic infant with a reactive cry. Dysmorphic facial features included hypertelorism, anteverted nares, Cupid's bow upper lip, gingival hypertrophy, ogival palate, and low-set ears. Delayed dentition was also observed, consistent with the dental manifestations commonly reported in malignant infantile osteopetrosis. Prominent frontal scalp veins were observed secondary to macrocephaly.

Ophthalmological examination demonstrated bilateral divergent strabismus associated with horizontal nystagmus, findings that may represent early manifestations of cranial nerve involvement secondary to skull base sclerosis. Formal ophthalmological evaluation for optic nerve compression was recommended; however, it had not yet been completed at the time of reporting. Similarly, formal audiological assessment was not available at the time of reporting. Given the known risk of sensorineural hearing loss resulting from auditory nerve compression in malignant infantile osteopetrosis, audiometry was recommended as part of the patient's ongoing multidisciplinary follow-up. Axial hypotonia was evident on neurological assessment. Abdominal examination revealed hepatosplenomegaly, and no peripheral lymphadenopathy was identified.

Investigations

Laboratory Findings

Serial complete blood counts confirmed persistent bicytopenia characterized by normocytic normochromic anemia and thrombocytopenia associated with leukocytosis and lymphocytic predominance, suggestive of impaired medullary hematopoiesis secondary to osteopetrosis. An infectious or inflammatory etiology was excluded based on normal C-reactive protein levels, negative blood cultures, and the absence of fever throughout the clinical evaluation

Radiological Findings

**Figure 1:**
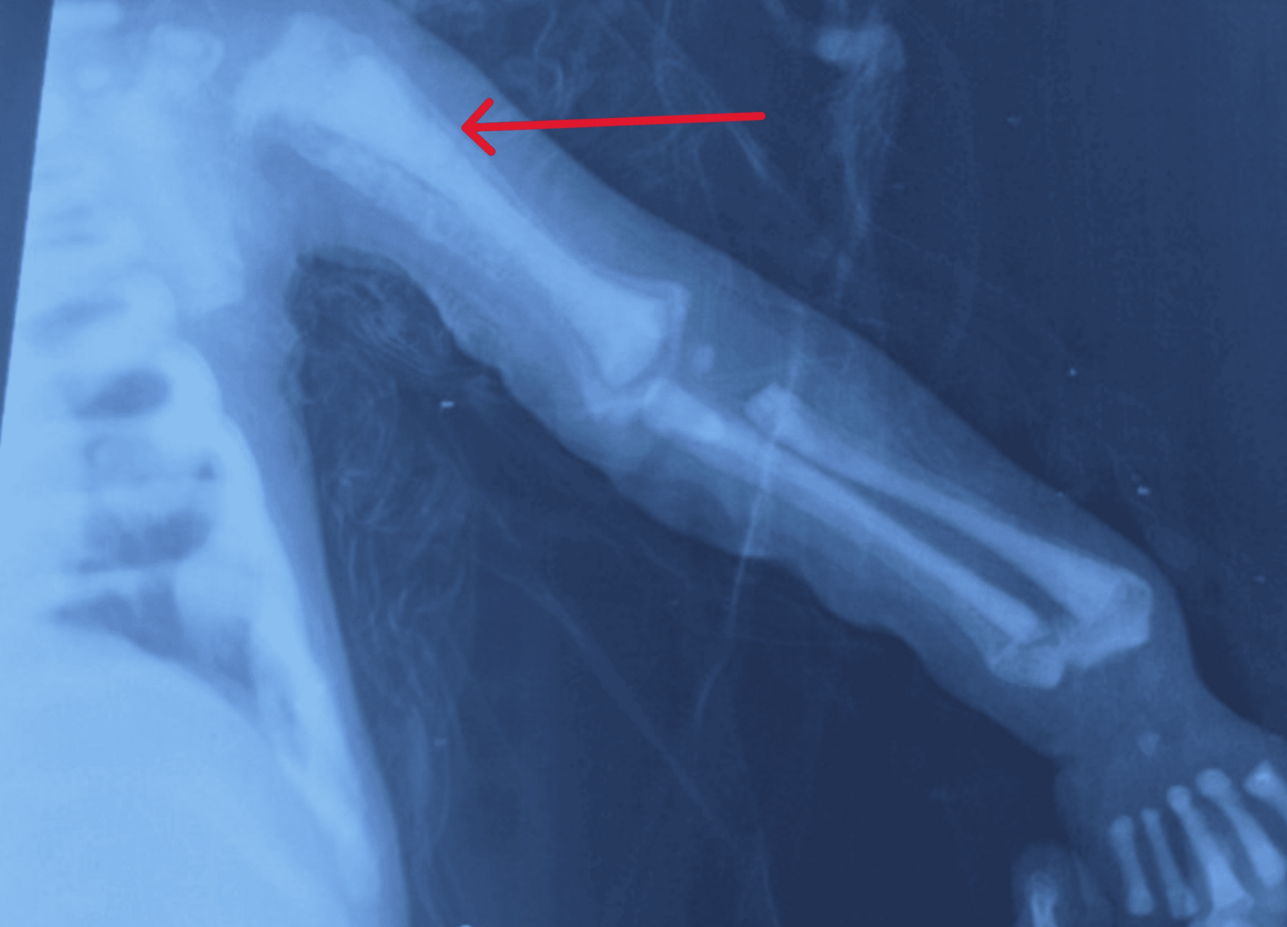
Plain radiograph of the upper limb showing diffuse osteosclerosis and obliteration of the medullary cavity Plain radiograph of the upper limb (humerus, radius, and ulna) demonstrating diffuse osteosclerosis with complete obliteration of the medullary cavity and loss of corticomedullary differentiation. A "bone-within-bone" (endobone) appearance is visible, a classic radiological hallmark of osteopetrosis. The red arrow indicates the area of medullary cavity obliteration in the humerus, consistent with malignant infantile osteopetrosis.

Conventional radiographs of the upper and lower limbs, hands, feet, and axial skeleton demonstrated classical features of malignant infantile osteopetrosis. Diffuse and homogeneous osteosclerosis involved all visualized osseous structures, with a marked increase in bone density and near-complete obliteration of the medullary cavities (Figure 1).

Metaphyseal flaring of the long bones produced the characteristic "Erlenmeyer flask" deformity. Cortical differentiation was lost, with cortex and medullary spaces appearing uniformly dense and opaque.

**Figure 2:**
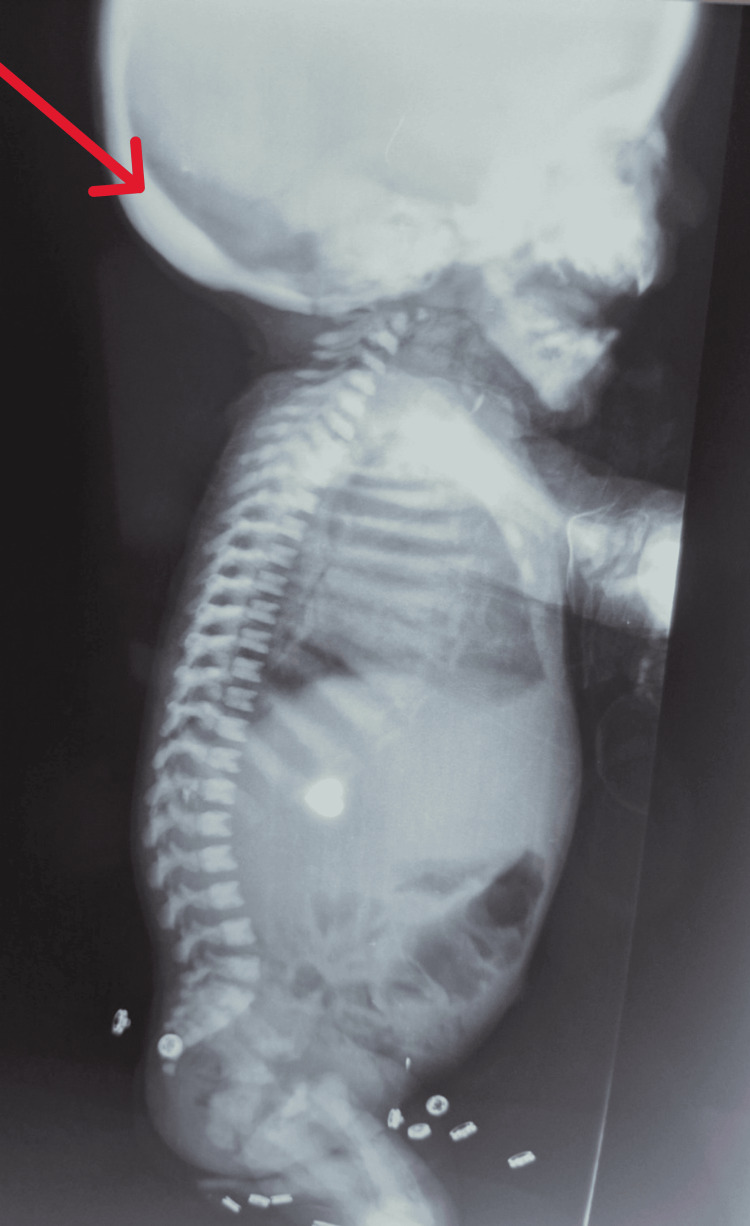
Lateral radiograph of the skull and spine showing pronounced calvarial thickening and homogeneous hyperdensity Lateral radiograph of the skull demonstrating pronounced calvarial thickening and homogeneous hyperdensity with narrowing of the cranial foramina, consistent with malignant infantile osteopetrosis. The arrow indicates the area of calvarial thickening.

Lateral skull radiographs revealed marked calvarial thickening and diffuse hyperostosis with narrowing of cranial foramina (Figure 2), a finding associated with an increased risk of cranial nerve compression.

Brain computed tomography (CT) demonstrated bilateral frontoparietal cortico-subcortical atrophy. Although the original CT images were unavailable for inclusion, this finding was documented in the official radiological report. Neurological involvement was suspected based on the presence of axial hypotonia, strabismus, and nystagmus, together with the reported CT findings, suggesting early cranial nerve and central nervous system involvement in the setting of malignant infantile osteopetrosis.

Abdominal ultrasonography confirmed hepatosplenomegaly, consistent with extramedullary hematopoiesis. No structural abnormalities of the urinary tract were identified.

Genetic Analysis

Whole-exome sequencing (WES) was performed on genomic DNA extracted from peripheral blood at a certified molecular genetics laboratory. Analysis identified a homozygous pathogenic variant in the TCIRG1 gene (NM_006019.4): c.1897C>T, predicted to result in a premature stop codon at amino acid position 633 (p.Gln633Ter).

This stop-gain variant is predicted to cause loss of normal protein function through nonsense-mediated mRNA decay (NMD) or generation of a severely truncated protein. The variant was absent from the Genome Aggregation Database (gnomAD v4.1.0) and has not previously been reported in affected individuals.

The variant was classified as pathogenic based on the American College of Medical Genetics and Genomics/Association for Molecular Pathology (ACMG/AMP) guidelines, supported by (1) PVS1 (null variant in a gene, where loss of function is a known disease mechanism); (2) PM2 (absence from population databases); and PP4 (highly specific clinical phenotype consistent with TCIRG1-associated osteopetrosis).

The identified molecular defect was fully consistent with the clinical and radiological diagnosis of autosomal recessive malignant infantile osteopetrosis (OMIM #259700). Full variant details are summarized in Table 1.

**Table 1:** Molecular characteristics of the identified TCIRG1 variant TCIRG1: T-cell immune regulator 1; ACMG: American College of Medical Genetics and Genomics; gnomAD: Genome Aggregation Database; PVS1: null variant in gene where LOF is disease mechanism; PM2: absent from population databases; PP4: phenotype highly specific for disease

Parameter	Details
Gene	TCIRG1
Transcript	NM_006019.4
cDNA change	c.1897C>T
Protein change	p.Gln633Ter
Genomic position (GRCh38)	chr11:68049672 C>T
Variant type	Stop-gain
Predicted consequence	Nonsense-mediated mRNA decay/protein truncation
Zygosity	Homozygous
gnomAD v4.1.0 frequency	Absent - not observed
Previously reported	No - novel variant
ACMG classification	Pathogenic
ACMG criteria applied	PVS1, PM2, PP4
Disease association	Malignant infantile osteopetrosis (OMIM: 259700)
Secondary findings	None identified

Parental segregation analysis has been recommended to confirm heterozygous carrier status in both parents.

Management and follow-up

Following molecular confirmation of the diagnosis, the patient was referred to a specialized hematopoietic stem cell transplantation (HSCT) program, which currently represents the only curative treatment for TCIRG1-related malignant infantile osteopetrosis.

Supportive management includes transfusion support for symptomatic anemia and thrombocytopenia, nutritional optimization with caloric supplementation, neurological monitoring, and regular ophthalmological surveillance for optic nerve compression and visual impairment.

Corticosteroid therapy was discontinued after confirmation of the genetic diagnosis because of the absence of sustained clinical or hematological benefit.

The family received comprehensive genetic counseling regarding the autosomal recessive mode of inheritance, the 25% recurrence risk for future pregnancies, and the availability of prenatal and preimplantation genetic testing.

At the most recent follow-up, the patient remains under hematological surveillance and is currently undergoing evaluation as a candidate for HSCT.

## Discussion

We report a patient with MIOP carrying a previously unreported homozygous TCIRG1 variant, c.1897C>T (p.Gln633Ter). This variant has not been described in affected individuals and is absent from major population databases, including gnomAD. Its identification expands the mutational spectrum of TCIRG1-associated osteopetrosis and further illustrates the marked allelic heterogeneity that characterizes this disorder [1].

The TCIRG1 gene encodes the a3 (ATP6i) subunit of the vacuolar H+-ATPase proton pump, a key component of osteoclast-mediated bone resorption. This proton pump is responsible for acidification of the extracellular resorption lacuna, an essential process for dissolution of hydroxyapatite crystals and degradation of mineralized bone matrix [4]. The c.1897C>T variant introduces a premature termination codon at amino acid position 633 (p.Gln633Ter), within the transmembrane region of the protein. As a stop-gain variant, it is predicted to result in loss of normal protein function through nonsense-mediated mRNA decay (NMD) or production of a severely truncated protein lacking critical transmembrane domains. This mechanism is entirely consistent with the established loss-of-function disease mechanism underlying TCIRG1-related osteopetrosis [8].

The pathogenicity of this variant is further supported by ACMG/AMP classification criteria [9], including PVS1 (predicted loss-of-function variant in a gene where loss of function is a known disease mechanism), PM2 (absence from population databases), and PP4 (highly specific clinical and radiological phenotype consistent with TCIRG1-associated osteopetrosis).

The clinical presentation of our patient corresponds to the severe end of the MIOP spectrum. The early onset of hematological abnormalities at four months of age, including normocytic normochromic anemia and thrombocytopenia, reflects progressive replacement of hematopoietic marrow by osteosclerotic bone and subsequent myelophthisis, one of the hallmark manifestations of MIOP [2,3]. The associated leukocytosis with lymphocytic predominance likely reflects compensatory extramedullary hematopoiesis secondary to bone marrow failure, further supported by the presence of hepatosplenomegaly.

Radiological findings were characteristic of MIOP and included diffuse osteosclerosis, obliteration of medullary cavities, metaphyseal Erlenmeyer flask deformities, and marked calvarial thickening. In the appropriate clinical context, these features strongly suggest the diagnosis; however, molecular confirmation remains essential for definitive diagnosis, accurate genetic counseling, prenatal diagnosis, and family screening.

Neurological complications represent a major cause of morbidity in MIOP. Progressive thickening of the skull base may lead to narrowing of cranial nerve foramina, resulting in optic neuropathy, hearing impairment, facial nerve palsy, and hydrocephalus [6]. The presence of strabismus and nystagmus in our patient may represent early manifestations of cranial nerve involvement, highlighting the importance of early recognition and prompt therapeutic intervention before irreversible neurological injury occurs.

HSCT remains the only curative treatment for TCIRG1-related MIOP and should be performed as early as possible, ideally within the first six months of life and before the onset of irreversible neurological complications, as outcomes are significantly improved when transplantation is undertaken at an earlier age [3,10,11]. Our patient is currently undergoing evaluation as a candidate for HSCT. Early molecular diagnosis is therefore crucial, as it facilitates timely referral to transplantation centers and optimizes the chances of long-term survival and preservation of neurological function.

Several studies have demonstrated that truncating TCIRG1 variants are consistently associated with severe infantile osteopetrosis. Pangrazio et al. reported numerous nonsense, frameshift, and splice-site variants distributed throughout the gene, all leading to a comparable severe phenotype [12]. Similarly, Wu et al. described a Chinese infant harboring compound heterozygous truncating TCIRG1 variants with a clinical presentation closely resembling that observed in our patient [13]. In North Africa, El-Kamah et al. reported multiple pathogenic TCIRG1 variants in Egyptian families and demonstrated that biallelic loss-of-function variants are a major cause of severe osteopetrosis in populations with high rates of consanguinity [14]. Collectively, these observations strongly support the pathogenic role of the novel c.1897C>T (p.Gln633Ter) variant identified in our patient.

Finally, this case highlights the diagnostic value of WES in infants from consanguineous families presenting with unexplained bicytopenia, failure to thrive, developmental abnormalities, and suggestive skeletal findings. Finally, this case highlights the diagnostic value of WES in infants from consanguineous families presenting with unexplained bicytopenia, failure to thrive, developmental abnormalities, and suggestive skeletal findings. Consanguineous marriages represent approximately 20-27% of unions in Morocco, with first-cousin marriages being the most frequent type [15]. This high rate of consanguinity contributes to an increased prevalence of rare autosomal recessive disorders in the region. In such settings, early implementation of genomic testing can substantially shorten the diagnostic odyssey, guide clinical management, facilitate access to curative therapies, and improve genetic counseling for affected families.

## Conclusions

We report a Moroccan infant with MIOP caused by a novel homozygous TCIRG1 pathogenic variant, c.1897C>T (p.Gln633Ter). This variant has not previously been reported in affected individuals, thereby expanding the mutational spectrum of TCIRG1-associated osteopetrosis.

This case highlights the importance of considering MIOP in infants presenting with failure to thrive, cytopenias, hepatosplenomegaly, developmental delay, and characteristic radiological findings of generalized osteosclerosis. Early molecular diagnosis through comprehensive genomic testing is essential to establish the diagnosis, guide genetic counseling, and facilitate timely referral for HSCT, which remains the only curative treatment. Furthermore, this report underscores the significant contribution of consanguinity to the burden of rare autosomal recessive disorders in North African populations and emphasizes the indispensable role of next-generation sequencing in achieving an accurate diagnosis and expanding current knowledge of disease-causing variants.
